# Congee Containing Carotenoids-Enriched Functional Ingredient from Tomato Improves Cognition, Serum α-Synuclein, Monoaminergic Function, and Gut-Brain Axis Functions in the Elderly Volunteers

**DOI:** 10.3390/life15071093

**Published:** 2025-07-11

**Authors:** Jintanaporn Wattanathorn, Supaporn Muchimapura, Wipawee Thukham-mee, Terdthai Tong-un

**Affiliations:** 1 Department of Physiology, Faculty of Medicine, Khon Kaen University, Amphoe Muaeng, Khon Kaen 40002, Thailand; supmuc@kku.ac.th (S.M.); meewep@gmail.com (W.T.-m.); terdthai@kku.ac.th (T.T.-u.); 2 Research Institute for High Human Performance and Health Promotion, Khon Kaen University, Amphoe Muaeng, Khon Kaen 40002, Thailand

**Keywords:** carotenoids, tomato, cognition, gut-brain axis, monoaminergic, elderly

## Abstract

Owing to the need for a brain supplement targeting neuroprotection against age-related brain disorders and the known effect of carotenoids on brain function, we aimed to assess the effect of consuming carotenoid-rich functional congee for 8 weeks on cognitive function and age-related serum biomarkers. Both males and females (60–70 years old) were randomly assigned to consume either placebo or carotenoid-rich functional congee containing dried tomato powder at doses of 200 and 400 mg per day. Cognitive function, working memory, and serum biomarkers including alpha-synuclein and PARK7, together with serum oxidative stress parameters and neurotransmitters, were assessed prior to consumption and every 4 weeks throughout the study period. The contents of *Lactobacillus* and *Bifidobacterium spp*. in feces were also determined. Both doses of carotenoid-enriched congee enhanced cognitive function (P300), but only low doses improved working memory and decreased the activity of MAO-A and serum alpha-synuclein. The high-dose congee-treated group exhibited an increase in the density of *Lactobacillus spp*. in feces. Taking all data together, the carotenoid-enriched congee enhances cognitive function and working memory. The mechanisms may partly involve the increase in monoaminergic function, while the modulation of the gut–brain axis may require further confirmation.

## 1. Introduction

Global aging is occurring rapidly. It has been reported that around 10% of the global population are aged 65 or older, approximately 800 million people [1]. With advancing age, the risks for developing neurodegenerative diseases such as Alzheimer’s disease and Parkinson’s disease (PD) increase [2]. These maladies are regarded as leading causes of physical and cognitive disability, resulting in a high negative impact on the socio-economic burden [3]. At present, all neurodegenerative diseases are clinically unmanageable. No drugs can reverse the neurodegeneration [4]. Due to the limitations of current therapy, prevention has been considered [5].

Among neurodegenerative diseases, Parkinson’s disease is regarded the second most common. The neurodegeneration of dopaminergic neurons in the substantia nigra in Parkinson’s disease is also attributed to excess oxidative stress and the accumulation of protein misfolding in the insoluble inclusions in neurons known as Lewy bodies [6], resulting in both motor disorders and cognitive impairment. It has been reported that the main composition observed in the Lewy bodies, the main hallmark of Parkinson’s disease, is α-synuclein, a presynaptic neuronal protein [7]. Recent findings demonstrate that the levels of α-synuclein in cerebrospinal fluid (CSF) and in serum are associated with the severity of motor symptoms [8] and cognition [9,10]. Therefore, it has been used as a target for the treatment and prevention of Parkinson’s disease [11,12,13]. In addition to the synucleinopathy and dopamine deficit mentioned earlier, cholinergic deficit [14,15] has also been reported. Due to the aforementioned changes, they are also targeted for treatment.

Recently, it has been demonstrated that carotenoids, the natural colorants in various plants, possess antioxidants, neuroprotection, and cognitive-enhancing effects [16,17,18,19]. Carotenoid-enriched plants such as tomato or *Solanum lycopersicum* Mill improve brain susceptibility to oxidative stress and inflammation in hypertensive rats [20]. It also suppresses oxidative stress and cholinesterase in the rat brain [21]. A mixture of tomato and lemon extracts can synergistically improve cognition and neurogenesis in aged mice [22]. Tomato seed extract also mitigates oxidative stress, mitochondrial dysfunction, and neurotoxicity induced by rotenone in a rat model of Parkinson ‘s disease [23]. In addition, it has been demonstrated that the intake of a tomato-enriched diet can protect against neurotoxicity induced by 6-hydroxydopamine in the substantia nigra [19]. Our previous work also revealed that tomato pomace also protects against stroke [18]. Moreover, encapsulated tomato fruit extract improves psychomotor function in healthy sports players [24]. Owing to the potential benefits of tomato on the biological activities related to neuroprotection and cognitive enhancement mentioned earlier, it has been suggested that tomato could potentially be used as the functional ingredient for improving cognitive function and protecting against synucleinopathies such as Parkinson’s disease. Since rice is the main food used in Asian countries, carotenoid-enriched functional congee was developed. To the best of our knowledge, there has been no scientific evidence concerning the effect of carotenoid-enriched functional congee on cognitive function and the main changes in the pathophysiology of Parkinson’s disease such as oxidative stress, neurotransmitter changes, and serum biomarkers of Parkinson’s disease until now. Therefore, this study aimed to elucidate this issue.

## 2. Materials and Methods

### 2.1. Preparation of Carotenoid-Rich Functional Congee

**Figure 1:**
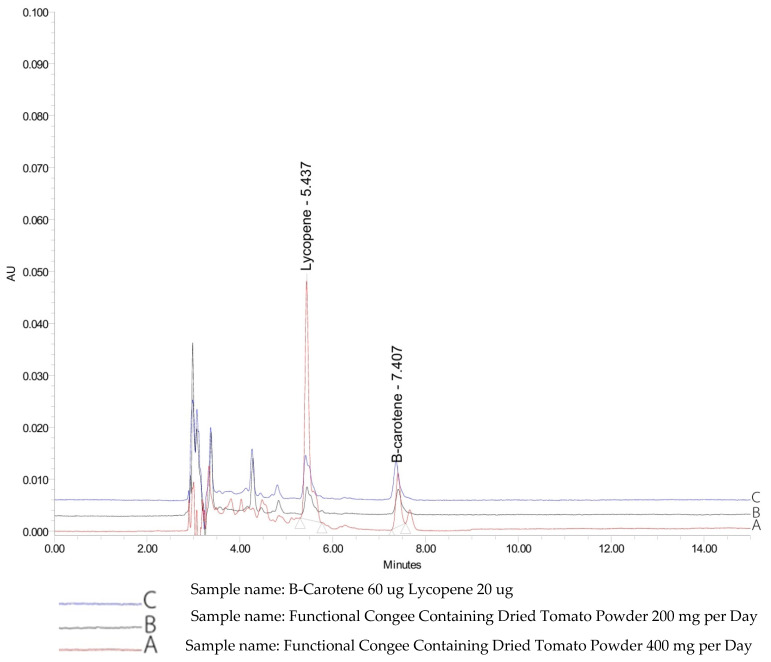
Fingerprint chromatograms showing the contents of carotenoid-enriched congee including lycopene and β-carotene at doses of 200 (A2) and 400 (A3) mg/serving.

Tomato or *S. lycopersicum* was supplied from an organic farm in Nongkhai province, After screening for contamination, it was prepared as tomato powder to serve as the functional ingredient. In brief, the tomato was washed, cut into small pieces, and boiled for 15 min. Then, the mixture was filtered and dried as powder in an oven at 50 °C for 10–14 h. In this study, placebo contained rice flakes, maltodextrin, pea fiber, salmon, and food additive. The carotenoid-enriched congee was prepared similarly to the placebo except the dried tomato powder was added at doses of 200 and 400 mg. The final products of placebo and the tested products showed the same appearance. It was found that the carotenoid-enriched congee at the doses of 200 (A2) and 400 (A3) mg contained lycopene at concentrations of 17 ± 0.001 and 18 ± 0.001 µg/g and contained beta-carotene at concentrations of 25 ± 0.001 and 32 ± 0.001 µg/g, respectively. In addition, the low-dose carotenoid-enriched congee also contained ferulic acid at a concentration of 0.266 ± 0.192 µg/g. The carotenoid profile of the carotenoid-enriched product was determined by using a high-performance liquid chromatography (HPLC) system (Waters Corporation, Milford, MA, USA) consisting of an in-line degasser AF, Waters 515 HPLC pump (pump control module II), Rheodyne injector with a sample loop of 20 µL, Waters 2998 photodiode array detector (USA), and Poroshell 120 EC-C18 column (250 × 4.6 mm id, 4.0 µm). Acetonitrile:2-propanol:ethyl acetate (40:40:20 *v*/*v*/*v*) was used as the mobile phase, and an isocratic elution was used to maintain a constant mobile phase composition throughout the running process. The results are shown in Figure 1.

### 2.2. Study Design

A single-center, 3-arm, randomized, double-blind, placebo-controlled, parallel study was deployed following the guidance of the International Conference of Harmonization (ICH) for Good Clinical Practice (GCP) and in compliance with the Declaration of Helsinki and its further amendments. All the study processes were reviewed and approved by the Khon Kaen University Ethical Committee on Human Research (HE631539), and it was registered at the Thai Clinical Trials Registry (TCTR 20201020003). Written informed consent of each participant was provided before participating in this study.

Sample size

This study is a pilot and proof of concept. The sample size was calculated based on the cognition. According to this study, the significance level was set at 5% (one-sided) to obtain 90% power [25,26,27,28] and the acceptable sample size per group, N, was set to 20 [28]. However, we also calculated the sample size by using the cognition obtained from the event-related potential as the primary outcome [29], and the obtained sample size was 15. We also used a withdrawal sample size of around 25% (around 4 persons). Therefore, the sample size according to our calculation was around 19/group. However, we used around 20 samples per group, which was the recommended sample size.

Inclusion and exclusion criteria

The following inclusion criteria were used: healthy male and female volunteers aged between 60 and 70 years old who could comply with the visit dates during the study period and had good communication skills in the Thai language; had a body mass index (BMI) ranging between 18.5 and 25 kg/m^2^, and a stable body weight during the 1 month before participating in this study; had a blood pressure not exceeding 140/90 mmHg and fasting blood sugar not exceeding 126 mg/d.L; had no history of head trauma, drug abuse, or addiction, and were smoking no more than 10 pieces/day; and were not athletes and did not exercise more than 3 times/week or participate in other projects. The following exclusion criteria were used: aspartate transaminase or AST > 64 U/L, and/or alanine transaminase or ALT > 50 U/L; afflicted with any serious physical and psychological illness during the study period; and using drugs or any substance(s) that could interfere with cognitive function. Subjects who consumed probiotics were also excluded.

Study protocol

A total of 92 male and females between the ages of 60 and 70 years old were recruited to participate in this study. After the interview and physical examination, there were 63 subjects who were allowed to participate. However, one subject in the placebo group withdrew from the study due to migration to another province. In addition, one subject from the experimental group at a dose of 200 mg per day withdrew due to nausea after 1 week of administration, and one subject from the experimental group at a dose of 400 mg per day withdrew due to a car accident. Therefore, 60 (20/group) total subjects participated in all visits of this study.

**Figure 2:**
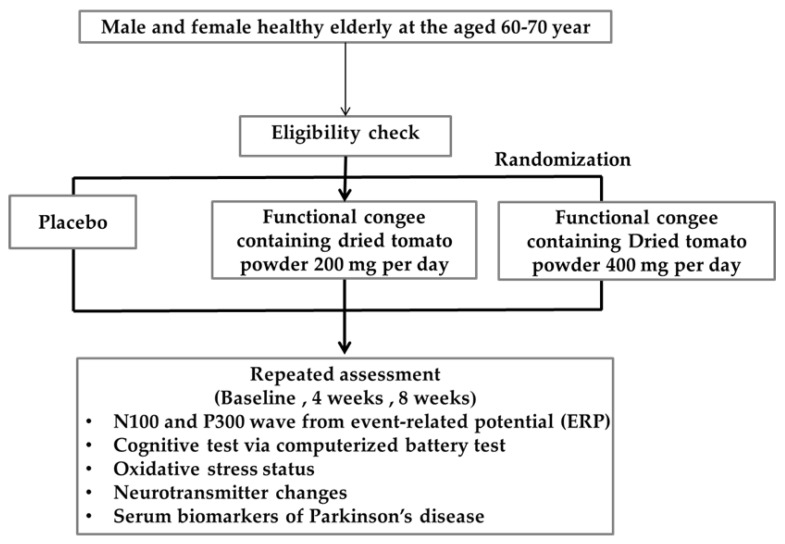
Flow chart of study design overview.

The applied procedures are demonstrated in Figure 2. In brief, all participants were randomized and divided into the placebo and carotenoid-enriched functional congee-treated groups at doses of 200 and 400 mg per day, respectively. Cognitive function was assessed by the event-related potential and was used as the primary outcome. The secondary outcomes were working memory, oxidative stress status, neurotransmitter changes, and serum biomarkers including alpha synuclein and PARK7. The safety parameters included hematological and clinical chemistry parameters and were also measured as secondary outcomes. All parameters were measured prior to and during the intervention at 4 and 8 weeks.

### 2.3. Event-Related Potential

In this study, we used the international 10/20 system to measure 10-min brain activity via an auditory odd ball paradigm of event-related potential. In brief, a 40-channel electrode cap (Neuroscan, Inc., Sterling, Herndon, VA, USA) with a scalp Ag-AgCl electrode and a reference connected to the earlobes was applied. Each subject was exposed to auditory stimuli at both non-targeted (650 Hz) and targeted (1 KHz) frequencies with an interstimulus interval of 1250 msec and an intensity of around 60 dB at approximately 85 and 15% of the total stimuli through headphones (NordicNeuroLab, Milwaukee, WI, USA). The subjects had to react to distinguish the targeted and non-targeted stimuli and select the corresponding button in front of them. Brain wave analysis was performed by evaluating the amplitude and latency of N100 (negative peak between 65 and 135 msec) and P300 (positive peak between 280 and 375 msec) at the Cz location which showed the optimum peak patterns [29], which were analyzed using Scan 4.3 analysis software (Neuroscan, Inc., Sterling, Herndon, VA, USA).

### 2.4. Working Memory Assessment

Power of attention, continuity of attention, quality of memory, and speed of memory were assessed using a computerized battery test consisting of the simple reaction time, choice reaction time, digit vigilance task, word recognition, picture recognition, numeric working memory, and spatial memory task force test [29,30,31]. All subjects were exposed to a 20-min computer presentation via VGA color monitors. All responses were recorded via two-button (YES/NO) response boxes.

Word Presentation

A set of 15-word recognition tests with an interstimulus interval of 1 s was presented to subjects. Each subject had to retrieve the word sequence in the correct order.

Picture Presentation

A set of 20 photographic images was provided to each subject via the monitor at a rate of 1 every 3 s, with a stimulus duration of 1 s. Then, the subjects had to retrieve information regarding the presentation.

Simple Reaction Time

During the test, a set of 15 stimuli with an interstimulus interval of 1 s was provided to each subject, who had to perform a quick response by pressing the word “yes” every time the word “yes” was displayed on the monitor.

Digit Vigilance Task

Each subject was exposed to a series of digits presented in the center of the screen at a rate of 80 /min. During this process, a target digit was randomly selected and constantly displayed on the right of the monitor screen. They had to quickly match the digit in the series with the target digit and press the “yes” button as quickly as possible. The percentage of accuracy response and the reaction time (milliseconds) were detected.

Choice Reaction Time

A total of 50 trials of the words “no” or “yes” were provided randomly with equal probability (interstimulus interval of 1 s) to each subject via the screen, and the subjects had to perform a quick response by pressing the corresponding button. Both reaction time (millisecond) and percentage of accuracy response (%) were measured.

Spatial Working Memory

Each participant was exposed to a motor screen displaying a picture of a house containing nine windows, four of which were lit. The subjects had to match the positions of the illuminated windows with a subsequent presentation of 36 similar pictures as quickly as possible by pressing the “yes” or “no” button. Both reaction time and percentage of accuracy response were monitored.

Numeric Working Memory

According to this test, a set of 30 probe digits was matched with the 5-digit stimuli previously exposed by pressing the “yes” or “no” response button as quickly as possible. Each subject was exposed twice with different stimuli and probe digits. The mean of both reaction times and accuracy of responses were detected.

The response times of simple reaction time, choice reaction time, and digit vigilance reflected the power of attention, whereas the accuracy responses of choice reaction time and digit vigilance tasks reflected the continuity of attention. In addition, the accuracy responses and response times of spatial working memory, numeric working memory, word recognition, and picture recognition reflected the quality and speed of memory, respectively.

### 2.5. Blood Collection and Preparation

A tube without ethylenediaminetetraacetic acid (EDTA) was used for collecting blood from the antecubital vein. Then, serum was prepared and used to determine all biochemical parameters.

### 2.6. Biochemical Assessments

#### 2.6.1. Acetylcholinesterase (AChE) Activity Assessment

In this study, we used the colorimetric method [29] to measure AChE activity in the serum. In brief, a 96-well microplate containing a mixture of 25 µL of 15 mM ATCI, 75 µL of 3 mM DTNB, and 50 µL of 50 mM Tris-HCl, pH 8.0, containing 0.1% bovine serum albumin (BSA), was allowed to react with the tested substance at a volume of 25 µL/well. After mixing, they were incubated for 5 min, and the absorbance at 415 nm was read (iMark™ Microplate Absorbance Reader, Bio-Rad Company, Hercules, CA, USA). Following this step, 10 µL of acetylcholine thiochloride (ACTI) was added and incubated for 3 min. Then, the absorbance of the mixture was measured at 415 nm [29,32] and the activity was calculated according to the equation below, expressed as mmol/min/g protein.

AChE activity = (rA/1.36 × 10^4^) × 1/(20/230)C

(rA = the difference in absorbance/minute, C = protein concentration of brain homogenate) 

#### 2.6.2. Monoamine Oxidase (MAO) Assessment

Both monoamine oxidase type A and type B (MAO-A and MAO-B, respectively) were measured based on the principle that the amino substrate was converted by MAO into aldehyde, amine, and hydrogen peroxide, which then oxidized 4-aminoantipyrine in the presence of peroxidase. The oxidized 4-aminoantipyrine condensed with vanillic acid to give a red quinoneimine dye, which could then be measured at 498 nm. For the assessment of MAO-A, 50 µL of sample and 50 µL of 500 nM pargyline were mixed and incubated for 30 min. Then, 200 µL of 500 µM P-tyramine was added, and the absorbance was measured at 490 nm. In order to assess MAO-B activity, all procedures were performed except that 50 µL of 500 nM chogyline was replaced with 500 nM pargyline [33,34].

#### 2.6.3. Assessments of Serum Biomarkers of Parkinson’s Disease

The serum biomarkers of Parkinson’s disease were assessed by using the ELISA kit of human alpha-synuclein and PARK7 (EH359RB, Thermo-Fisher Scientific, Waltham, MA, USA). An aliquot of serum at a volume of 50 µL was added to a 96-well microplate coated with antibody against alpha-synuclein or antibody against PARK7 and mixed with 50 µL of antibody cocktail. After mixing, the mixture was incubated for 1 h and washed. Following this step, 100 µL of TMB Development Solution was added to enhance the reaction and incubated for 10 min. At the end of the incubation period, a 100 µL stop solution was added. The absorbance at 450 nm was determined for both parameters.

#### 2.6.4. Determination of Oxidative Stress Markers

Malondialdehyde (MDA) levels were measured using a reaction of thiobarbituric acid reactive substances (TBARSs) according to the method of Ohkawa et al. [35]. The activities of the main scavenger enzymes that include superoxide dismutase (SOD), catalase (CAT), and glutathione peroxidase (GSH-Px) were assessed spectrophotometrically via the suppression of nitroblue tetrazolium (NBT) reduction [36], a reduction in H_2_O_2_ absorbance [37], and the formation of NADP+ [33,34], respectively. This process is described in detail in our previous studies [38,39].

### 2.7. Assessment of Lactobacillus spp. and Bifidobacterium spp.

The contents of *Lactobacillus spp*. and *Bifidobacterium spp*. in the stool sample were analyzed within 8 h after collection. An aliquot of feces was diluted in phosphate buffer (1 g:9 mL), homogenized 5 times with the aseptic technique, and prepared as a serial dilution at concentrations ranging between 10^−1^ and 10^−7^. An aliquot of a feces sample from each dilution at a volume of 100 µL was spread on the surface of agar plates and incubated at 37 °C for 24 h under anaerobic conditions. Then, the *Lactobacullus* spp. and *Bifidobacteriumspp.* colonies were counted and expressed as colony-forming unit (CFU)/g of feces.

### 2.8. Statistical Analysis

All data are expressed as mean ± SD. The analysis was performed by using per-protocol analysis. The statistical analysis was performed via repeated-measurement analysis of variance. A non-parametric test was used for the analysis of data that failed to show a normal distribution. In all statistical comparisons, differences with *p*-values <0.05 were considered significant.

## 3. Results

### 3.1. Demographic Data, Vital Signs, Body Weight, Height, and Body Mass Index (BMI)

The demographic data, vital signs, body weight, and body mass index (BMI) of the volunteers in this study are shown in Table 1. It was found that the average age of the subjects in this study were 64.09–64.38 years old. No significant difference in vital signs including body temperature, heart rate, respiration, and blood pressure among groups were observed. Body weight, height, and BMI also failed to show significance differences among groups.

### 3.2. Effect of Functional Congee Containing Dried Tomato Powder on Cognitive Function

The baseline data of brain waves via the event-related potential of subjects are shown in Table 2. It was found that there were no significant differences in N100 and P300 components among the three different groups at baseline in this study. After 4 weeks of intervention, subjects who consumed the functional congee containing dried tomato powder at doses of 200 and 400 mg per day showed a reduction in latency of P300 at the Cz location (*p* < 0.05 and *p* < 0.01, respectively, compared to the placebo-treated group). However, this change disappeared when the consumption period was prolonged to 8 weeks. In addition, the amplitude of N100 at the Fz location of the subjects who consumed the functional congee containing dried tomato powder at a dose of 200 mg per day significantly increased after 4 and 8 weeks of consumption (*p* < 0.05 and *p* < 0.05, respectively, compared to the placebo group).

The effect of the functional congee containing dried tomato powder on four domains of working memory is shown in Table 3. Prior to the administration of the functional congee containing dried tomato powder, there were no significant differences among the three groups of subjects in this study. The current data revealed that the subjects who consumed the functional congee containing dried tomato powder at a dose of 200 mg per day during the 4-week consumption period exhibited a significant increase in the %accuracy in word recognition tasks, and this change was still present after the 8-week consumption period (*p* < 0.05 all, compared to the placebo group). In addition, subjects who consumed the functional congee containing dried tomato powder at a dose of 200 mg per day during the 8-week consumption period also exhibited an increase in percent accuracy in spatial memory (*p* < 0.05, compared to the placebo group). These data revealed that the carotenoid-enriched functional congee in this study significantly improved the speed and quality of memory.

### 3.3. Effect of Functional Congee Containing Dried Tomato Powder on Biochemical Changes

In order to probe for the possible underlying changes in cognitive function, the serum changes in oxidative stress markers including malondialdehyde (MDA) level and the serum activities of the main scavenger enzymes such as superoxide dismutase (SOD), catalase (CAT), and glutathione peroxidase (GPx) were determined. It was found that there were no significant differences in any parameters mentioned above prior to the consumption of the assigned substances and throughout the 8-week study period, as demonstrated in Table 4.

The effects of the functional congee containing dried tomato powder on the alterations in the neurotransmitters playing pivotal roles in cognitive function such as acetylcholine and monoamine and Parkinson’s biomarkers such as alpha-synuclein and PARK7 were also investigated. It was found that after 4 weeks of consumption, the subjects who consumed the functional congee containing dried tomato powder at doses of 200 and 400 mg per day exhibited a significant decrease in alpha synuclein. During the 8-week consumption period, a reduction in MAOA was observed in the subjects who consumed the functional congee containing dried tomato powder at both doses. By contrast, a reduction in alpha-synuclein was observed only in the subjects who consumed the functional congee containing dried tomato powder at a dose of 400 mg per day (*p* < 0.05 all, compared to the placebo group), and a significant reduction in this parameter was observed in both experimental groups during the 4-week consumption period, as shown in Table 5.

### 3.4. Changes in Lactobacillus and Bifidobacterium spp.

Prior to the consumption of the carotenoid-rich functional congee, there were no significant differences in *Lactobacillus* and *Bifidobacteriumspp.* Although there were no significant differences in the densities of *Lactobacillus* and *Bifidobacteriumspp.* in the feces of the placebo and experimental group, it was found that subjects who consumed a high dose of carotenoid-rich functional congee exhibited a significant increase in *Lactobacillus spp*. in the feces (*p* < 0.01, compared to placebo-treated group), whereas no significant changes were observed in the placebo- and low-dose-treated groups. The current data failed to show significant changes in *Bifidobacteriumspp.* in all groups, as shown in Table 6.

## 4. Discussion

The current data showed that the possible active ingredient of the functional congees was carotenoids, especially beta-carotene and lycopene. The volunteers who consumed the carotenoid-rich functional congee containing dried tomato powder at a dose of 200 mg/day for 4 weeks showed a significant increase in N100 amplitude, and this change was still present after the 8-week consumption period. This change indicates the increase in attention toward the stimuli [40]. After the 4-week consumption period, the volunteers who consumed the functional congee containing dried tomato powder at both doses used in this study showed a reduction in P300 latency, but this change disappeared when the consumption was prolonged to 8 weeks. In addition, consumption of the functional congee containing dried tomato powder at a dose of 200 mg/day also improved the percent accuracy of the word recognition test after both consumption periods, but an improvement in response time of volunteers who consumed the functional congee at this dose showed a significant reduction only after the 8-week consumption period. Moreover, a significant improvement in the percent of accuracy response in spatial memory in this volunteer group was also detected after 8 weeks of consumption. The alterations in P300 latency suggested an improvement in the cognitive processing capability [36], while the improvement in the computerized battery test observed in this study suggested an improvement in both quality and speed of working memory [29,30,31]. Owing to the report regarding the association between dementia and protein misfolding [7,9,10], we also assessed the level of serum misfolding proteins such as alpha-synuclein and PARK7 in order to probe for the possible underlying mechanism in memory enhancement. Interestingly, our findings revealed a reduction in MAO-A in subjects who consumed the developed functional congee at both doses after 8 weeks of consumption. The reduction in alpha synuclein was also present in the volunteers who consumed the developed functional congee at both doses after 4 weeks of consumption, and this change was still observed in the volunteers who consumed the high dose of the developed product. Since several lines of evidence demonstrated the role of monoamine transmitters in N100 brain waves or attention [41,42], and P300 brain waves or cognitive processes [43], we suggested that the improvement in attention and cognitive process observed in this study might be partly associated with the reduction in MAO-A, which in turn increased monoaminergic activity [41,42,43,44]. Since monoamine was also reported to modulate both spatial and non-spatial working memory, we suggest that the improvement in word recognition and spatial memory [45] assessed by the computerized battery test in this study might partly relate to the aforementioned changes. Beyond the crucial role of the monoaminergic system, alpha synuclein was also demonstrated to be associated with numerous changes in synucleinopathy disorders such as Parkinson’s disease. The elevation in plasma alpha-synuclein level was associated with cognitive decline in Parkinson’s disease [46], whereas an elevation in alpha-synuclein gave rise to neurodegeneration in Parkinson’s disease [13], and this substance was used as a serum marker for the early diagnosis of Parkinson’s disease [9]. Therefore, the reduction in serum alpha synuclein observed in this study might indirectly relate to the reduction in neurodegeneration, and result in the decrease in the risk of Parkinson’s disease and dementia.

A recent study during this decade revealed that *Lactobacillusspp.* such as *L. plantarum* PS128 could attenuate the neurodegeneration of monoaminergic such as dopaminergic neurons in an animal model of Parkinson’s disease [47]. In addition, *L. plantarum* DP189 could also decrease alpha synuclein, giving rise to the mitigation of monoaminergic neurodegeneration in a mice model of Parkinson’s disease [48]. Although the study just mentioned also proposed the modulatory role of oxidative stress on this neuroprotective effect, our study failed to demonstrate a significant change in oxidative stress markers in this study. This discrepancy between our study and the mentioned study might be partly associated with the different species used in experiments and the difference in the assessed sample, because we determined the serum level of oxidative markers, whereas the previous study determined the brain’s oxidative markers. Since oxidative stress markers were under the influence of many pathways and many organ changes, the lack of a significant difference in oxidative markers might also partly attribute to the aforementioned phenomena. The improvement in the cognitive process observed in the high-dose-treated group may be partly associated with the improved gut microbiome by increasing *Lactobacillus spp.,* giving rise to the improvement in gut–brain axis function, resulting in the improvement in neurodegeneration in the circuit involving cognitive processes.

The developed functional congee containing dried tomato powder used in this study was rich in carotenoids such as lycopene and beta-carotene, which previously demonstrated the suppression effect of MAO [49], neuroprotection against synuclein toxicity [50,51], and cognitive function enhancement [16,52]. In addition, carotenoids such as beta lycopene also increased the content of *Lactobacillus spp*. [52,53,54,55]. Therefore, all improvements presented in this study after the consumption of the functional congee containing dried tomato powder may be associated with carotenoids presented in the developed product. Since consuming food during the experimental period could induce confounding errors on cognition and memory in the subject, we also assessed the amount, frequency, and types of food consumed during the study period by using the food frequency questionnaire (FFQ), which showed a significant reduction in food, rice, and vegetables containing polyphenols during the study period (data are shown in Supplementary Table S1). Therefore, it is less likely to exert a confounding effect on cognition and working memory. Although the comparison of *Lactobacillusspp.* density in the feces of subjects who consumed a high dose of congee showed a significant difference when compared to baseline data, that of the placebo group showed no significant difference in this parameter. The positive modulation effect on Lactobacillus spp. still requires further confirmation with a larger sample size and more details on the link between the positive modulation effect of the high-dose congee and the *Lactobacillusspp.* density in feces, and the link between *Lactobacillusspp.* density in the feces and gut–brain axis stimulation, together with the link between gut–brain axis stimulation and cognition and working memory.

Our findings demonstrated that the improved P300 induced by the carotenoid-enriched congee disappeared when the consumption was prolonged to 8 weeks, while the alterations in working memory, MAO-A, and α-synuclein are still present. These findings suggested that cognitive processing failed to show a closed relationship with the mentioned parameters. The possible explanation for this event might be associated with the multi-factor involvement of the mentioned parameters, especially the P300 brain waves which occurred as a result of the summation of electrical potential in the brain area that was measured and was under the influence of many transmitters such as cholinergic, noradrenergic, dopaminergic, serotoninergic, and GABAergic systems [56]. Owing to the influences of various transmitters beyond the monoaminergic system and the summation of electrical potential of the neuronal pool of the brain circuit in that area, no closed relationships between brain wave activity and the parameters mentioned above were observed.

In this study, the developed functional congee containing dried tomato powder failed to show a dose-dependent response. This phenomenon may occur partly because the tested congee contains numerous ingredients which can produce interactions among various ingredients. In addition, the observed parameters also involve multiple factors; therefore, it is not possible to show a linear relationship with the concentration of the tested congee.

The strength of this study is the integration of culturally tailored functional foods with the validated biomarkers of psychophysiology parameters such as brain waves from an auditory oddball paradigm of event-related potential (ERP), as well the battery of cognitive tests—the validated tools for assessing cognition and working memory in clinical trials [57,58,59,60,61]. However, there are many limited points that require further exploration such as the effects of the developed novel food on multi-omics changes to provide a precise understanding of the underlying mechanisms which consist of numerous complex biological pathways of the intervention. The weak point of this study appears to be the determination of some parameters which are not covered all changes of oxidative stress. In this study, MDA was used as an indicator to reflect lipid peroxidation status [60] which, in turn, reflects an increase in oxidative stress [62]. Unfortunately, MDA is not sensitive enough, and another marker which is more sensitive such as F2-isoprostanes should also be assessed to confirm the effect of the novel food on the lipid peroxidation process [63], which in turn indicates oxidative stress status. Furthermore, other indices such as 8-hydroxydeoxyguanosine (8-OHdG) and protein carbonyl, the parameters reflecting the attack of oxidative stress on DNA and protein [64], should also be investigated in order to reflect the overall oxidative stress status. Another weak point in this study appears to be associated with the determination of the change in gut microbiota. The parameters used in this study such as lactic acid-producing bacteria (LAB) and the density of *Lactobacillus* and *Bifidobacterium spp*. are not sufficient to reflect the overall changes in the gut microbiota communities. The precise understanding regarding the crosstalk between food, the gut microbiota, and its host requires more advanced techniques such as functional metagenomics [65]. Moreover, further research regarding the mechanism linking gut–brain stimulation with the alteration of gut microbiota induced by an intervention of the novel food used in this study is still essential to provide the underlying mechanisms. This study was carried out in healthy volunteers, and no criteria were present for lumbar puncture and the determination of the biomarkers in cerebrospinal fluid (CSF). Therefore, the determinations of the synuclein, PARK-7, and oxidative stress markers in CSF are less likely to be possible due to misconduct.

The present data clearly demonstrate the cognitive enhancement potential of a carotenoid-enriched congee in the elderly. Since this study focuses on the prevention or slowing down of cognitive decline in healthy volunteers, we found that at least 1 month of consumption of the novel functional food can produce a significant increase in cognition in the elderly volunteers. No information is available regarding how long the novel supplement must be consumed to maintain or enhance cognition and how long this effect lasts for after its cessation. Therefore, a larger sample size and longer treatment duration such as a larger cohort and longer follow-up study must be used in future studies to generalize these findings. Another limitation of this study appears to be associated with the lack of data on the serum level of carotenoids. The correlation between carotenoid content and cognitive function, the changes in various neurotransmitters playing the roles of P300 generation and modulation, and an assessment of alpha-synuclein together with the link between brain plasticity, attention, memory, and *Lactobacillusspp.* will provide the precise underlying mechanism of cognitive change induced by the carotenoid-rich functional congee.

Taking everything together, we suggest that the reduction in serum biomarkers of Parkinson’s disease and the improvement in attention, cognitive function, and working memory in this study may relate partly to the carotenoids such as β-carotene and lycopene presented in the functional congee containing dried tomato powder, which may improve alpha synuclein and the monoaminergic system. The high dose of congee also exerted the role via the modulation of the growth of *Lactobacillusspp.* , which may stimulate the gut–brain axis. However, this modulation effect requires further exploration. The limitation of this study occurs partly due to the lack of carotenoid levels in the serum of subjects. In addition, there was no detailed determination of the underlying mechanisms regarding brain plasticity and the modulation of *Lactobacillusspp.* growth. A further exploration of the correlation between carotenoid content and cognitive function, the changes in various neurotransmitters playing the roles of P300 generation and modulation, and the assessment of alpha-synuclein together with the link between brain plasticity, attention, memory, and *Lactobacillusspp.* will provide the precise underlying mechanism of cognitive change induced by the carotenoid-rich functional congee.

This study provides interesting information about the application of food as the strategy to protect against brain dysfunction such as cognitive decline, working memory impairment, and age-related neurodegenerative diseases. The potential brain benefits mentioned earlier can be obtained from not only tomato but also other carotenoid-enriched foods such as watermelons, pink grapefruits, papayas, red guavas, apricots, and red bell peppers. Accumulative lines of evidence also demonstrate that many foods such as *Hericium* mushrooms, a traditional medicinal mushroom, and a plant in many species such as Leguminosae can also provide brain benefits [66,67,68,69]. Therefore, preventive medicine that applies foods as the protective strategy is one of the potential health-promoting strategies.

## 5. Conclusions

This study clearly demonstrates that the consumption of a carotenoid-rich functional congee containing dried tomato powder at doses of 200 and 400 g/day can improve cognitive function and working memory in elderly volunteers. This suggests the potential brain benefits of carotenoid-enriched foods. The mechanisms appear to be partly associated with an improvement in protein misfolding such as alpha synuclein and an increase in monoaminergic function through the suppression of MAO-A. High doses of the developed product can also increase *Lactobacillusspp.* in the gut community, may modulate the gut–brain axis, and improve brain plasticity, leading to an improvement in attention. However, the latter point requires further confirmation. Since the data failed to show a dose-dependent effect, the suggested dose for cognitive and memory enhancements may be 200 mg/day. In addition, based on the role of alpha synuclein as the serum marker of Parkinson’s disease, this product also shows the potential to protect against Parkinson’s disease. However, further research is still needed to confirm the mentioned benefits and gain a precise understanding of the detailed mechanisms. Another interesting point from this study is that the application of lycopene and β-carotene-enriched functional ingredients for promoting cognition and protecting against the protein misfolding of alpha synuclein may also be applied with other raw materials such as pink guava, watermelon, and papaya, and will increase the value of not only tomato but also other carotenoid-rich fruits mentioned earlier.

## Figures and Tables

**Table 1:** Demographic data, vital signs, body weight, height, and body mass index (BMI) of subjects prior to intervention and at 4 and 8 weeks of treatment. Data are shown as mean ± SD.

Parameter	Placebo	Functional Congee Containing Dried Tomato Powder 200 mg per Day	Functional Congee Containing Dried Tomato Powder 400 mg per Day
Age (year)	64.09 ± 4.00	64.38 ± 3.60	64.19 ± 3.09
		(*p* = 0.580)	(*p* = 0.668)
Gender (Male/Female)	(1/20)	(3/17)	(3/17)
Body Temperature (°C)			
Baseline	36.11 ± 0.32	35.99 ± 0.45 (*p* = 0.632)	36.09 ± 0.37 (*p* = 0.922)
4-week	36.23 ± 0.50	36.28 ± 0.41 (*p* = 0.824)	36.32 ± 0.41 (*p* = 0.582)
8-week	36.04 ± 0.40	36.23 ± 0.53 (*p* = 0.366)	36.17 ± 0.37 (*p* = 0.648)
Heart Rate (beats/min)			
Baseline	70.59 ± 5.97	69.42 ± 7.88 (*p* = 0.706)	68.33 ± 8.56 (*p* = 0.209)
4-week	71.95 ± 9.33	72.10 ± 8.06 (*p* = 0.834)	70.05 ± 10.36 (*p* = 0.619)
8-week	71.90 ± 9.05	69.80 ± 9.35 (*p* = 0.505)	67.95 ± 7.77 (*p* = 0.183)
Respiratory Rate (breaths/min)			
Baseline	17.18 ± 1.05	17.10 ± 1.14 (*p* = 0.815)	17.33 ± 0.97 (*p* = 0.616)
4-week	17.86 ± 0.85	17.50 ± 1.10 (*p* = 0.341)	17.60 ± 0.94 (*p* = 0.344)
8-week	18.38 ± 1.32	18.15 ±1.30 (*p* = 0.392)	18.25 ± 0.97 (*p* = 0.560)
Systolic BP (mmHg)			
Baseline	126.31 ± 14.96	126.76 ± 8.77 (*p* = 0.408)	130.00 ± 10.01 (*p* = 0.634)
4-week	122.38 ± 13.61	126.30 ± 11.33 (*p* = 0.441)	121.00 ± 22.38 (*p* = 0.705)
8-week	126.24 ± 15.90	125.70 ± 11.44 (*p* = 0.744)	126.50 ± 12.40 (*p* = 0.845)
Diastolic BP (mmHg)			
Baseline	70.81 ± 10.72	73.04 ± 6.54 (*p* = 0.349)	74.52 ± 10.30 (*p* = 0.263)
4-week	71.95 ± 9.38	75.00 ± 10.29 (*p* = 0.473)	72.40 ± 9.59 (*p* = 0.969)
8-week	74.05 ± 10.20	76.40 ± 8.17 (*p* = 0.284)	77.45 ± 9.51 (*p* = 0.187)
Body Weight (kg)			
Baseline	57.12 ± 7.59	60.40 ± 7.86 (*p* = 0.628)	62.90 ± 9.16 (*p* = 0.599)
4-week	56.89 ± 7.68	60.21 ± 8.00 (*p* = 0.124)	60.84 ± 9.72 (*p* = 0.137)
8-week	56.89 ± 7.68	60.54 ± 7.99 (*p* = 0.100)	61.41 ± 9.79 (*p* = 0.088)
Body Height (cm)			
Baseline	154.27 ± 5.12	156.09 ± 6.62 (*p* = 0.392)	156.23 ± 6.73 (*p* = 0.242)
4-week	157.27 ± 5.11	156.24 ± 6.79 (*p* = 0.424)	156.24 ± 6.73 (*p* = 0.242)
8-week	154.00 ± 5.08	156.05 ±6.79 (*p* = 0.345)	155.80 ± 6.59 (*p* = 0.255)
BMI (kg/m^2^)			
Baseline	24.01 ± 3.08	24.84 ± 3.27 (*p* = 0.174)	25.89 ± 4.41 (*p* = 0.117)
4-week	22.91 ± 5.99	24.79 ± 3.42 (*p* = 0.113)	23.84 ± 7.11 (*p* = 0.444)
8-week	24.01 ± 3.26	24.90 ± 3.21 (*p* = 0.112)	25.38 ± 4.59 (*p* = 0.179)

**Table 2:** The effects of the functional congee containing dried tomato powder on the cognitive function of the elderly and middle-aged volunteers assessed by event-related potential (N = 20/group). Data are expressed as the mean ± SD.

Event-Related Potentials			Before Consumption	After 4 Weeks of Consumption	After 8 Weeks of Consumption
Fz	N100 Latency (ms)	Placebo	105.04 ± 2.73	107.52 ± 1.49	103.43 ± 2.67
		Functional congee containing dried tomato powder 200 mg per day	107.33 ± 1.59 (*p* = 0.903)	108.10 ± 1.41 (*p* = 0.865)	106.81 ± 2.28 (*p* = 0.336)
		Functional congee containing dried tomato powder 400 mg per day	107.42 ± 1.54 (*p* = 0.789)	105.72 ± 1.65 (*p* = 0.471)	104.78 ± 2.50 (*p* = 0.692)
	N100 Amplitude (μV)	Placebo	5.08 ± 0.755	6.57 ± 1.08	6.31 ± 1.44
		Functional congee containing dried tomato powder 200 mg per day	7.78 ± 0.74 (*p* = 0.056)	9.44 ± 1.00 * (*p* = 0.030)	10.22 ± 1.71 * (*p* = 0.032)
		Functional congee containing dried tomato powder 400 mg per day	7.63 ± 0.88 (*p* = 0.071)	8.12 ± 1.20 (*p* = 0.481)	6.57 ± 1.35 (*p* = 0.647)
	P300 Latency (ms)	Placebo	337.54 ± 7.41	350.40 ± 5.94	352.54 ± 4.34
		Functional congee containing dried tomato powder 200 mg per day	346.42 ± 6.76 (*p* = 0.408)	352.45 ± 3.84 (*p* = 0.568)	358.20 ± 7.20 (*p* = 0.760)
		Functional congee containing dried tomato powder 400 mg per day	323.71 ± 9.96 (*p* = 0.422)	343.16 ± 7.29 (*p* = 0.104)	350.38 ± 4.45 (*p* = 0.963)
	P300 Amplitude (μV)	Placebo	9.83 ± 1.02	11.68 ± 1.88	15.68 ± 2.59
		Functional congee containing dried tomato powder 200 mg per day	9.97 ± 1.40 (*p* = 0.680	8.75 ± 1.42 (*p* = 0.245)	10.29 ± 1.73 (*p* = 0.152)
		Functional congee containing dried tomato powder 400 mg per day	8.88 ± 1.22 (*p* = 0.593)	10.74 ± 1.84 (*p* = 0.539)	14.60 ± 1.79 (*p* = 0.913)
Cz	N100 Latency (ms)	Placebo	108.52 ± 2.86	108.00 ± 1.64	105.93 ± 2.82
		Functional congee containing dried tomato powder 200 mg per day	106.85 ± 1.49 (*p* = 0.174)	108.10 ± 2.12 (*p* = 0.610)	107.29 ± 1.76 (*p* = 0.971)
		Functional congee containing dried tomato powder 400 mg per day	106.85 ± 1.91 (*p* = 0.052)	105.11 ± 2.33 (*p* = 0.480)	106.61 ± 2.13 (*p* = 0.959)
	N100 Amplitude (μV)	Placebo	4.84 ± 0.77	6.56 ± 1.29	7.22 ± 1.18
		Functional congee containing dried tomato powder 200 mg per day	7.96 ± 1.21 (*p* = 0.061)	8.87 ± 1.32 (*p* = 0.151)	8.46 ± 1.13 (*p* = 0.471)
		Functional congee containing dried tomato powder 400 mg per day	7.84 ± 1.09 (*p* = 0.051)	7.47 ± 0.93 (*p* = 0.190)	6.67 ± 0.82 (*p* = 0.863)
	P300 Latency (ms)	Placebo	339.09 ± 8.56	355.70 ± 6.06	332.80 ± 12.34
		Functional congee containing dried tomato powder 200 mg per day	345.28 ± 7.03 (*p* = 0.990)	345.25 ± 6.29 * (*p* = 0.036)	350.93 ± 6.83 (*p* = 0.203)
		Functional congee containing dried tomato powder 400 mg per day	315.61 ± 11.49 (*p* = 0.054)	333.16 ± 11.08 ** (*p* = 0.009)	346.33 ± 4.28 (*p* = 0.704)
	P300 Amplitude (μV)	Placebo	8.49 ± 1.08	10.73 ± 1.47	13.14 ± 1.37
		Functional congee containing dried tomato powder 200 mg per day	7.82 ± 1.35 (*p* = 0.372)	6.44 ± 1.48 (*p* = 0.123)	9.81 ± 1.76 (*p* = 0.486)
		Functional congee containing dried tomato powder 400 mg per day	6.56 ± 1.10 (*p* = 0.110)	9.94 ± 1.77 (*p* = 0.640)	11.51 ± 2.28 (*p* = 0.828)

* *p* < 0.05; compared to the placebo group; ** *p* < 0.01; compared to the placebo group.

**Table 3:** The effects of the functional congee containing dried tomato powder on the percent changes in working memory in the elderly and middle-aged volunteers assessed by the computerized battery test (N = 20/group). Data are expressed as the mean ± SD.

Cognitive Domains	Test Items	Treatment Group	Baseline Data	%Change in the Parameters from Baseline	
				4-Week	8-Week
Word Recognition	Response time	Placebo	2081.01 ± 229.09	−6.93 ± 12.19	17.41 ± 10.29
		Functional congee containing dried tomato powder 200 mg per day	1625.36 ± 128.11	−33.59 ± 7.08 (*p* = 0.060)	−42.76 ± 21.86 * (*p* = 0.035)
		Functional congee containing dried tomato powder 400 mg per day	1400.05 ± 75.37	−15.84 ± 6.82 (*p* = 0.754)	−30.66 ± 3.70 (*p* = 0.375)
	%Accuracy	Placebo	76.06 ± 3.16	5.76 ± 2.47	6.67 ± 2.81
		Functional congee containing dried tomato powder 200 mg per day	81.42 ± 2.97	26.63 ± 8.69 * (*p* = 0.052)	33.08 ± 10.84 * (*p* = 0.155)
		Functional congee containing dried tomato powder 400 mg per day	82.22 ± 3.31	11.56 ± 2.80 (*p* = 0.229)	13.87 ± 4.30 (*p* = 0.457)
		Placebo	1770.77 ± 144.49	−10.25 ± 5.92	−24.94 ± 6.12
	Response time	Functional congee containing dried tomato powder 200 mg per day	1493.25 ± 90.96	−23.68 ± 3.83 (*p* = 0.068)	−26.00 ± 4.21 (*p* = 0.715)
Picture Recognition		Functional congee containing dried tomato powder 400 mg per day	1241.06 ± 69.91	10.59 ± 5.07 (*p* = 0.958)	−14.30 ± 5.52 (*p* = 0.220)
	%Accuracy	Placebo	76.36 ± 2.85	11.82 ± 3.59	8.96 ± 4.86
		Functional congee containing dried tomato powder 200 mg per day	84.52 ± 12.73	7.03 ± 2.25 (*p* = 0.505)	11.72 ± 6.45 (*p* = 0.885)
		Functional congee containing dried tomato powder 400 mg per day	81.19 ± 2.12	11.10 ± 2.97 (*p* = 0.948)	8.25 ± 3.05 (*p* = 0.969)
Simple reaction	Response time	Placebo	961.08 ± 115.64	−10.79 ± 5.43	−10.90 ± 6.17
		Functional congee containing dried tomato powder 200 mg per day	751.94 ± 33.44	−13.13 ± 5.55 (*p* = 0.754)	−15.48 ± 6.90 (*p* = 0.715)
		Functional congee containing dried tomato powder 400 mg per day	738.27 ± 26.75	−7.58 ± 6.47 (*p* = 0.938)	−4.85 ± 4.70 (*p* = 0.481)
Digit vigilance	Response time	Placebo	653.35 ± 16.11	6.60 ± 2.18	7.15 ± 4.08
		Functional congee containing dried tomato powder 200 mg per day	687.62 ± 12.14	2.25 ± 2.13 (*p* = 0.090)	12.82 ± 14.78 (*p* = 0.112)
		Functional congee containing dried tomato powder 400 mg per day	692.83 ± 24.04	23.56 ± 11.89 (*p* = 0.297)	12.68 ± 8.93 (*p* = 0.230)
	%Accuracy	Placebo	88.75 ± 1.27	1.57 ± 1.57	0.72 ± 1.38
		Functional congee containing dried tomato powder 200 mg per day	90.04 ± 1.23	2.34 ± 1.34 (*p* = 0.552)	2.37 ± 1.37 (*p* = 0.256)
		Functional congee containing dried tomato powder 400 mg per day	89.31 ± 1.12	87.30 ± 1.57 (*p* = 0.647)	1.57 ± 1.84 (*p* = 0.876)
	Response time	Placebo	959.40 ± 52.72	−1.25 ± 4.40	−7.18 ± 5.18
Choice reaction time		Functional congee containing dried tomato powder 200 mg per day	922.68 ± 42.30)	−9.13 ± 5.85 (*p* = 0.179)	−8.44 ± 3.51 (*p* = 0.498)
		Functional congee containing dried tomato powder 400 mg per day	864.92 ± 47.51	−9.17 ± 5.61 (*p* = 0.285)	−2.68 ± 2.69 (*p* = 0.531)
	%Accuracy	Placebo	96.64 ± 0.65	0.53 ± 0.52	0.63 ± 0.64
		Functional congee containing dried tomato powder 200 mg per day	96.95 ± 0.54	−0.21 ± 0.91 (*p* = 0.256)	0.75 ± 0.48 (*p* = 0.439)
		Functional congee containing dried tomato powder 400 mg per day	96.04 ± 0.68	0.75 ± 1.02 (*p* = 0.916)	96.90 ± 0.60 (*p* = 0.636)
	Response time	Placebo	2174.57 ± 226.75	−9.47 ± 8.21	−17.70 ± 6.48
Spatial memory		Functional congee containing dried tomato powder 200 mg per day	1709.24 ± 147.42	−6.93 ± 12.17 (*p* = 0.735)	−18.69 ± 7.41 (*p* = 0.814)
		Functional congee containing dried tomato powder 400 mg per day	1531.76 ± 68.89	−3.83 ± 12.54 (*p* = 0.794)	−9.68 ± 7.27 (*p* = 0.309)
	%Accuracy	Placebo	71.84 ± 4.40	16.26 ± 6.39	20.05 ± 7.09
		Functional congee containing dried tomato powder 200 mg per day	78.96 ± 3.60	39.17 ± 9.78 (*p* = 0.130)	43.16 ± 8.67 * (*p* = 0.049)
		Functional congee containing dried tomato powder 400 mg per day	80.86 ± 3.33	34.02 ± 12.45 (*p* = 0.657)	43.08 ± 11.65 (*p* = 0.183)
Numeric working memory	Response time	Placebo	1536.97 ± 104.48	−12.07 ± 3.31	−18.19 ± 3.37
		Functional congee containing dried tomato powder 200 mg per day	1313.53 ± 57.73	−15.12 ± 4.23 (*p* = 0.449)	−15.02 ± 4.64 (*p* = 0.814)
		Functional congee containing dried tomato powder 400 mg per day	1212.64 ± 42.63	−11.00 ± 6.11 (*p* = 0.990)	−14.23 ± 4.58 (*p* = 0.335)
	%Accuracy	Placebo	86.21 ± 3.00	5.18 ± 5.47	4.44 ± 3.90
		Functional congee containing dried tomato powder 200 mg per day	88.57 ± 3.34	9.08 ± 5.61 (*p* = 0.592)	11.08 ± 4.48 (*p* = 0.353)
		Functional congee containing dried tomato powder 400 mg per day	87.92 ± 2.66	12.36 ± 7.36 (*p* = 0.520)	14.85 ± 13.87 (*p* = 0.824)

* *p* < 0.05; compared to the placebo group.

**Table 4:** The effects of the functional congee containing dried tomato powder on the serum changes in oxidative stress markers including malondialdehyde (MDA) level and the activities of the main scavenger enzymes such as superoxide dismutase (SOD), catalase (CAT), and glutathione peroxidase (GPx) in the serum (N = 20/group). Data are shown as the mean ± SD.

Parameters	Time	Placebo	Functional Congee Containing Dried Tomato Powder 200 mg per Day	Functional Congee Containing Dried Tomato Powder 400 mg per Day
MDA level (ng/mg.protein)	Baseline	0.04 ± 0.01	0.04 ± 0.01 (*p* = 0.725)	0.04 ± 0.01 (*p* = 0.347)
	4-week	0.03 + 0.01	0.03 ± 0.01 (*p* = 0.704)	0.03 ± 0.01 (*p* = 0.804)
	8-week	0.03 ± 0.01	0.03 ± 0.01 (*p* = 0.497)	0.03 ± 0.01 (*p* = 0.969)
SOD activity (U/mg.protein)	Baseline	1.60 ± 0.86	1.67 ± 0.91 (*p* = 0.917)	1.99 ± 0.48 (*p* = 0.159)
	4-week	1.57 ± 1.22	2.14 ± 1.68 (*p* = 0.686)	1.76 ± 1.68 (*p* = 0.262)
	8-week	1.97 ± 1.33	2.23 ± 0.41 (*p* = 0.735)	2.37 ± 0.41 (*p* = 0.876)
CAT activity (U/mg.protein)	Baseline	1.71 ± 0.51	1.59 ± 0.41 (*p* = 0.620)	1.47 ± 0.41 (*p* = 0.112)
	4-week	1.50 ± 0.41	1.85 ± 0.67 (*p* = 0.106)	1.69 ± 0.66 (*p* = 0.566)
	8-week	2.00 ± 0.71	2.06 ± 0.75 (*p* = 0.794)	1.83 ± 0.74 (*p* = 0.657)
GSH-Px activity (U/mg.protein)	Baseline	3.66 ± 1.48	3.25 ± 1.43 (*p* = 0.657)	3.51 ± 1.94 (*p* = 0.735)
	4-week	3.33 ± 0.98	3.49 ± 1.13 (*p* = 0.584)	3.70 ± 1.44 (*p* = 0.159)
	8-week	4.37 ± 1.50	4.30 ± 1.23 (*p* = 1.000)	4.65 ± 1.18 (*p* = 0.657)

**Table 5:** The effects of the functional congee containing dried tomato powder on the alterations in acetylcholinesterase (AChE), monoamine oxidase (MAO), monoamine oxidase type A (MAOA) and type B (MAOB), alpha-synuclein, and DJ-1 (PARK7) in serum (N = 20/group). Data are shown as the mean ± SD.

Parameters	Time	Placebo	Functional Congee Containing Dried Tomato Powder 200 mg per Day	Functional Congee Containing Dried Tomato Powder 400 mg per Day
AchE	Baseline	8.91 ± 2.14	8.47 ± 1.94 (*p* = 0.896)	8.72 ± 2.21 (*p* = 0.754)
(nmol/mg protein)	4-week	8.26 ± 2.13	9.20 ± 3.44 (*p* = 0.584)	9.16 ± 2.75 (*p* = 0.241)
	8-week	9.16 ± 2.13	9.46 ± 2.94 (*p* = 0.917)	8.73 ± 2.30 (*p* = 0.434)
MAO-A	Baseline	0.49 ± 0.15	0.46 ± 0.21 (*p* = 0.862)	0.46 ± 0.35 (*p* = 0.807)
(umol/h/mg protein)	4-week	0.48 ± 0.15	0.56 ± 0.23 (*p* = 0.335)	0.52 ± 0.56 (*p* = 0.057)
	8-week	0.65 ± 0.25	0.47 ± 0.32 * (*p* = 0.024)	0.47 ± 0.20 * (*p* = 0.032)
MAO-B	Baseline	0.62 ± 0.30	0.56 ± 0.33 (*p* = 0.322)	0.50 ± 0.22 (*p* = 0.251)
(umol/h/mg protein)	4-week	0.61 ± 0.27	0.60 ± 0.27 (*p* = 0.549)	0.64 ± 0.30 (*p* = 0.958)
	8-week	0.96 ± 0.22	0.97 ± 0.33 (*p* = 0.566)	0.94 ± 0.29 (*p* = 0.506)
Alpha-synuclein (pg/mL)	Baseline	35.77 ± 8.12	35.43 ± 6.21 (*p* = 0.896)	36.89 ± 7.79 (*p* = 0.814)
	4-week	50.69 ± 11.54	44.82 ± 6.21 * (*p* = 0.048)	43.63 ± 11.22 * (*p* = 0.015)
	8-week	53.23 ± 10.82	52.41 ± 11.98 (*p* = 0.597)	48.82 ± 11.06 * (*p* = 0.011)
PARK7 (ng/mL)	Baseline	1.12 ± 0.21	1.11 ± 0.20 (*p* = 0.958)	1.14 ± 0.21 (*p* = 0.774)
	4-week	1.10 ± 0.19	1.13 ± 0.21 (*p* = 0.938)	1.07 ± 0.20 (*p* = 0.382)
	8-week	1.06 ± 0.93	0.89 ± 0.22 (*p* = 0.735)	0.82 ± 0.20 (*p* = 0.449)

AChE = acetylcholinesterase inhibitor, MAO = monoamine oxidase, MAOA = monoamine oxidase type A, MAOB = monoamine oxidase type B, PARK7 = protein parkinsonism-associated deglycase. * *p* < 0.05; compared to the placebo group.

**Table 6:** The effects of the placebo and low- and high-dose carotenoid-rich functional congee on the contents of *Lactobacillus* and *Bifidobacteriumspp.* in feces (N = 20/group). Data are expressed as the mean ± SD.

Groups	Total Count of *Lactobacillusspp.* (Mean of CFU/mL)		Total Count of *Bifidobacteriumspp.* (Mean of CFU/mL)	
	Baseline	8-Week	Baseline	8-Week
Placebo	1.44 × 10^8^ ± 1.04 × 10^8^	2.82 × 10^7^ ± 2.23 × 10^6^ (*p* = 0.310)	1.35 × 10^6^ ± 4.43 × 10^4^	5.54 × 10^7^ ± 19.00 × 10^7^
Functional congee containing dried tomato powder 200 mg	4.50 × 10^7^ ± 3.21 × 10^7^	1.74 × 10^9^ ± 1.49 × 10^9^ (*p* = 0.291)	2.32 × 10^6^ ± 7.92 × 10^4^	1.16 × 10^6^ ± 5.19 × 10^6^
Functional congee containing dried tomato powder 400 mg	3.65 × 10^7^ ± 9.48 × 10^6^	3.54 × 10^8^ ± 6.17 × 10^7^ ## (*p* = 0.009)	2.32 × 10^6^ ± 2.54 × 10^4^	6.30 × 10^5^ ± 28.17 × 10^5^

## *p* < 0.01 compared to the baseline.

## Data Availability

The data presented in this study are available on request from the corresponding author.

## Supplementary Materials

The following supporting information can be downloaded at: https://www.mdpi.com/article/10.3390/life15071093/s1, The amount of food consumed per day during the study period assessed by using the Food Frequency Questionnaire (FFQ) is presented in Table S1.
