# Supplementary material for: Congee Containing Carotenoids-Enriched Functional Ingredient from Tomato Improves Cognition, Serum α-Synuclein, Monoaminergic Function, and Gut-Brain Axis Functions in the Elderly Volunteers
Source: Life (Basel). 2025 Jul 11;15(7):1093. doi: 10.3390/life15071093 (PMC12301000; doi:10.3390/life15071093)
Supplement: Supplementary file 1 [file life-15-01093-s001.zip › life-3687629-supplementary.pdf]

Table S1 Amount of consumed food /day during the experimental period (8 week-study period) of volunteers consumed placebo, and the functional congee containing dried tomato powder at the doses of 200, and 400 mg/day. Data were expressed as Mean±SEM * P<0.05; compared to placebo

Parameter	Treatment group	Placebo	Congee containing dried tomato powder 200 mg/day	Congee containing dried tomato powder 400 mg/day
**Rice**	**Baseline**	19.00 ± 0.78	19.19 ± 0.80 (p=0.975)	19.95 ± 0.54 (p=0.470)
	**4-week**	20.00 ± 0.54	19.20 ± 1.13 (p=0.848)	18.90 ± 1.10 (p=0.395)
	**8-week**	19.90 ± 0.69	20.10 ± 0.62 (p=0.955)	19.95 ± 0.55 (p=0.691)
**Meat**	**Baseline**	14.68 ± 1.24	17.28 ± 1.14 (p=0.144)	16.61 ± 1.188 (p=0.291)
	**4-week**	17.66 ± 1.33	16.00 ± 1.52 (p=0.712)	15.60 ± 1.38 (p=0.372)
	**8-week**	14.23 ± 1.64	15.40 ± 1.08 (p=0.772)	12.30 ± 1.31 (p=0.320)
**Egg**	**Baseline**	7.27 ± 1.32	3.76 ± 0.89 (p=0.066)	5.61 ± 0.95 (p=0.431)
	**4-week**	8.90 ± 1.58	5.45 ± 1.22 (p=0.141)	6.40 ± 1.32 (p=0.272)
	**8-week**	8.80 ± 1.16	7.70 ± 1.67 (p=0.444)	6.35 ± 1.11 (p=0.184)
**Milk**	**Baseline**	6.77 ± 1.37	3.04 ± 0.74 (p=0.102)	3.42 ± 1.02 (p=0.139)
	**4-week**	3.95 ± 1.31	2.80 ± 0.94 (p=0.692)	2.45 ± 0.94 (p=0.487)
	**8-week**	3.76 ±1.29	3.50 ± 1.29 (p=0.927)	1.75 ± 0.70 (p=0.341)
**Vegetable**	**Baseline**	17.59 ± 1.17	17.52 ± 4.91 (p=0.459)	14.90 ± 1.45 (p=0.114)
	**4-week**	17.23 ± 1.23	16.80 ± 0.94 (p=0.502)	17.10 ± 1.13 (p=0.714)

	**8-week**	15.33 ± 1.28	15.05 ± 1.05 (p=0.779)	12.95 ± 1.46 (p=0.259)
**Fruits**	**Baseline**	13.18 ± 1.58	10.66 ± 1.19 (p=0.166)	8.24 ± 1.30 *(p=0.032)
	**4-week**	14.61 ± 1.52	13.30 ± 1.43 (p=0.510)	11.60 ± 1.35 (p=0.147)
	**8-week**	10.19 ± 1.54	10.50 ± 1.48 (p=0.849)	7.70 ± 1.60 (p=0.245)
**Coconut Curry**	**Baseline**	1.95 ± 0.80	1.57 ± 0.52 (p=0.853)	2.09 ± 0.66 (p=0.482)
	**4-week**	1.00 ± 0.54	0.35 ± 0.35 (p=0.323)	0.35 ± 0.35 (p=0.323)
	**8-week**	1.42 ± 0.73	0.70 ± 0.70 (p=0.207)	1.05 ± 0.57 (p=0.780)
**Dessert**	**Baseline**	4.09 ± 1.15	2.28 ± 0.87 (p=0.184)	2.28 ± 0.66 (p=0.313)
	**4-week**	6.38 ± 0.98	1.95 ± 0.70 **(p=0.001)	2.85 ± 0.77 *(p=0.015)
	**8-week**	3.42 ± 0.97	1.05 ± 0.76 *(p=0.028)	0.70 ± 0.48 *(p=0.019)
**Snack**	**Baseline**	5.14 ± 1.60	1.66 ± 0.60 (p=0.214)	3.80 ± 1.18 (0.958)
	**4-week**	3.47 ± 1.07	1.95 ± 0.79 (0.316)	1.85 ± 0.85 (p=0.294)
	**8-week**	1.90 ± 0.94	2.65 ± 0.91 (p=0.307)	1.05 ± 0.57 (p=0.690)
